# Health Care Professionals’ Perspectives on Implementing Patient-Accessible Electronic Health Records in Primary Care: Qualitative Study

**DOI:** 10.2196/64982

**Published:** 2025-06-06

**Authors:** Irene Muli, Åsa Cajander, Isabella Scandurra, Maria Hägglund

**Affiliations:** 1 Participatory eHealth and Health Data Research Group Department of Women’s and Children’s Health Uppsala University Uppsala Sweden; 2 Department of Information Technology Uppsala University Uppsala Sweden; 3 Centre of Empirical Research in Information Systems School of Business Örebro University Örebro Sweden

**Keywords:** patient accessible electronic health record, implementation, consolidated framework for implementation research, primary care

## Abstract

**Background:**

Patients are increasingly being offered online record access (ORA) through patient-accessible electronic health records (PAEHRs), but implementation is often met with resistance from health care professionals (HCPs). Experiences from previous implementations may provide important insights into potential barriers and facilitators.** **

**Objective:**

This study aimed to investigate the factors influencing the implementation of the Swedish PAEHR system in primary care from the perspectives of HCPs.

**Methods:**

We conducted 14 semistructured interviews with a diverse group of HCPs shortly after the implementation of the Swedish PAEHR system. The interviews were analyzed using the Consolidated Framework for Implementation Research (CFIR) and content analysis, identifying key themes related to PAEHR implementation.

**Results:**

The analysis identified several potential factors influencing the implementation of the Swedish PAEHR system. According to the HCPs, the PAEHR system was *flawed* but also *flexible*. The HCPs described working in *a complex and imperfect organization*, which nonetheless had an *existing structure*, *support*, *and established communication* with patients. They also described *nondocumentation-related use of the electronic health record system.* Moreover, they reported dealing with a *complicated patient group* with varying needs and high expectations. The HCPs expressed that they worked in a patient-centered way and with *patient engagement*. The HCPs could see both the *advantages and disadvantages* of the PAEHR system and had some concerns. There were *mixed views of the extent of the change*, where some felt patient ORA would not affect their work at all and others expected a substantial impact. Some HCPs had experience using the PAEHR system themselves, while some *lacked knowledge and interest*. Furthermore, the implementation process was perceived as *long and uneventful*, with *fragmented communication*, where *existing communication activities were used*. The HCPs also reported receiving some information and education about PAEHRs outside the organization. The HCPs had limited awareness of how patients were introduced to the PAEHR system.

**Conclusions:**

This study underscores the importance of having a usable electronic health record system and addressing organizational issues, such as issues with the work environment, for optimal implementation of eHealth services such as the PAEHR system. It also highlights the importance of HCPs’ views and experiences with their patients, and their perceptions and attitudes toward the intervention. Additionally, this study stresses the importance of effective implementation processes and communication strategies for both HCPs and patients.

## Introduction

### Background

In the present health care landscape, patient-accessible electronic health records (PAEHRs) have been implemented in several countries worldwide [1-4]. With the European Health Data Space proposal, this transformation is set to accelerate further [5]. Yet, despite the reported benefits of patient online record access (ORA), including improved patient-provider relationships, improved medication adherence, and reduced anxiety [6], implementation has proven challenging in many contexts, not the least due to concerns and resistance from health care professionals (HCPs) [7-10].

HCPs have expressed several concerns regarding patient ORA [11-15]. For example, although they acknowledge some potential benefits of PAEHRs, they also believe that the records might be incomprehensible or that patients might misunderstand them due to the presence of medical language and potentially complex content. Concerns are that patients may become unnecessarily worried or make decisions based on misunderstanding that could cause harm. HCPs have also reported changing documentation practices to reduce the risk of confusion by writing notes more comprehensively and by omitting hypotheses [12,16], which may make the documentation less clinically useful [17]. In addition, HCPs have been concerned about the potential increase in their workload due to the implementation of ORA [8]. After implementation, HCPs have reported some increase in their workload [12,16] related to, for example, helping patients access PAEHRs and understand their content. In contrast, for some professional groups, there have been reports of a decrease in workload related to administering paper copies of electronic health records (EHRs) to patients [16].

Furthermore, HCPs have expressed concerns that the record’s content might worry patients [14,15]. After implementation, patients have reported experiencing distress from reading their records online [12,18]. In Sweden, according to a national survey study, 26% of participants worried about something they had read, and they usually searched for answers online, called the hospital, or raised their concerns at their next visit [18]. Another concern among HCPs is the safeguarding of vulnerable patient groups (eg, domestic abuse victims and at-risk children) [13,19]. Patients are, however, not as concerned about security and privacy and have not experienced many breaches [20-22].

While several studies have focused on attitudes and experiences of PAEHRs, few have investigated their implementation and how the implementation process could affect the concerns of HCPs. According to the Consolidated Framework for Implementation Research (CFIR), 5 domains are important to consider when studying the implementation of interventions: intervention characteristics, inner setting, outer setting, characteristics of the individuals involved, and the implementation process itself [23]. The framework can be used to both plan implementation projects and study successful and failed implementations. Considering the challenges and delays described in the implementation of patient ORA, it is important to deepen our understanding of the factors that may affect the introduction in different contexts, including HCP concerns. In this study, we used the CFIR to structure our analysis and help explain HCPs’ concerns and attitudes toward patient ORA and their experiences of the implementation in a Swedish primary care context.

In Sweden, the first patient ORA trial took place in a primary care center (PCC) in 1997 and had promising results [24], but implementation soon faced legal roadblocks, which took years to resolve [25]. In 2008, the new Patient Data Act allowed health care providers to give patients ORA, and implementation could resume. Scale-up was step-wise, and eventually, all patients in Region Uppsala gained access to PAEHRs in 2012. The local PAEHR system was integrated with the National Health Information Exchange infrastructure in 2015, and in 2016, 17 of the 21 Swedish regions were providing patients with access to their EHRs [25]. By 2018, all Swedish regions were providing patients with ORA.

Aspects, such as what health record information is accessible to patients, who can access it, and when is it made available, can vary from region to region and between health care providers [1-4,26,27]. Sweden has a national regulatory framework for patient ORA, first established in 2012 with mandatory and elective paragraphs [28]. Due to the legal framework’s flexibility, the 21 autonomous Swedish regions made different decisions on what information can be accessed, when it can be accessed, and who can access it. An updated national regulatory framework from 2016 promises more unification [29], but it is yet to be fully adopted. Individual HCPs have little control over the system and can, for example, not delay access to certain information. Health care providers can, however, opt to make specific keywords used in the EHR inaccessible to patients in PAEHRs to safeguard patients (eg, early hypotheses or concerns for domestic abuse) [30]. 

Swedish regions adopted different implementation strategies and faced various barriers [31]. In Uppsala, the first region to implement PAEHRs, HCPs were not involved, leading to resistance and negative impacts on implementation. Recognizing this, other regions engaged HCPs, influencing decisions such as restriction of patient access to EHR entries made after implementation [31]. Stakeholders globally emphasized that HCP and patient involvement and education, and institutional change strategies are key to successful implementation [7,32].

As HCPs play a critical role in the adoption of patient ORA, it is essential to further deepen our understanding of what factors, from their perspectives, influence successful implementation. This study, therefore, explores the implementation of the Swedish PAEHR system in primary care from the HCP perspective. Investigation of the factors influencing implementation might increase our understanding of important barriers and facilitators, offering valuable insights into the challenges and potential areas of improvement in PAEHR implementation.

To analyze the factors influencing the implementation of PAEHRs, we used the CFIR. The CFIR was chosen because it is a well-established and widely used framework in implementation science, providing a structured approach to identifying both barriers and facilitators across multiple levels of the health care system. Compared to other implementation frameworks, such as the Normalization Process Theory and the Theoretical Domains Framework, the CFIR offers a broader scope by incorporating individual, organizational, and contextual factors, making it particularly suitable for studying the adoption of digital health interventions in complex health care settings. The CFIR has previously been applied in research on eHealth implementation, including patient portals and EHRs, further supporting its relevance for this study [33].

### Aim

We aimed to investigate the factors influencing the implementation of the Swedish PAEHR system in primary care from HCPs’ perspectives.

## Methods

### Study Setting

The study was part of an investigation into the implementation of a PAEHR system in primary care and its usability and implications for HCPs. Swedish health care is mainly tax-funded, and most hospitals and PCCs are managed by regional councils [34]. Since the regions are autonomous, primary care can vary between regions. Both publicly and privately owned PCCs exist, and they are generally publicly funded. Most PCCs are team-based with a mix of different HCPs (eg, general practitioners [GPs], nurses, physiotherapists, and dietitians). The average national primary care visit count was 4.3 per person in 2009 [34]. Compared to the health care systems in other countries, the Swedish health care system struggles with waiting times, continuity of care, and support for chronically ill patients [35]. The PCC included in this study is located in the largest Swedish region, Region Stockholm, and has a mixed patient population with varying socioeconomic backgrounds. While it may not be representative of all PCCs in Sweden, it is an interesting center with a fairly large and diverse patient population and a common mix of health care and administrative staff.

The Swedish PAEHR system, also known as 1177 Journal, is accessible through the national patient portal [36]. Citizens in Region Stockholm had access to medical notes, lab results, diagnoses, and information about visits when the PAEHR system was launched (2016/2017). In 2023, they were provided access to maternity care, vaccination, and referral records, as well as log reports [37].

In Stockholm, over 73% of the population had good self-reported health, and the life expectancy was 82 years in 2014 [38].

### Recruitment of Study Participants

Study participants were recruited from an academic PCC, which has a special focus on education and research. Information about the study was distributed via email through the PCC manager as well as orally during workplace meetings. HCPs were eligible to participate in the study if they were involved in the clinical documentation process by writing clinical notes themselves in the EHRs (eg, nurses), dictating clinical notes (eg, physicians), or being involved in the transcription of clinical notes (eg, medical secretaries). A convenience sampling approach was used, and it was voluntary to participate. Nonparticipation was not analyzed.

### Data Collection

Data were collected through individual interviews. The interviews were conducted face-to-face at the PCC by the last author (MH) 6 months after the launch of the PAEHR system in Region Stockholm (2017). MH is a female researcher/lecturer with a PhD degree, who, at the time of the interviews, had extensive experience in qualitative research. The interviews were conducted using a semistructured interview guide developed by MH and the third author (IS). The interview guide was pilot-tested with 1 primary care physician, and minor changes were made to the order of questions and the terminology used. The interview guide is presented in Multimedia Appendix 1. The HCPs were interviewed about their experiences of the implementation process, which was the focus of this analysis, as well as the impact of the PAEHR system on the work environment [16] and what they would like to improve to increase the usability and usefulness of the PAEHR system [39]. The interviews were conducted in Swedish and lasted for 30 to 45 minutes. The interviews were audio recorded. 

Data saturation was not fully achieved due to challenges in recruiting study participants.

### Data Analysis Using the CFIR

The recorded interviews were transcribed and analyzed by the first author (IM) in discussion with the rest of the authors. The transcribed interviews were read and reread. The analysis was conducted in 2 steps using an abductive approach, that is, a combination of inductive and deductive analysis [40]. First, the analysis was conducted deductively using the CFIR, and the information was coded using the CFIR domains (intervention characteristics, inner setting, outer setting, characteristics of individuals, and implementation process) [23]. In this study, the CFIR was a fitting choice owing to its comprehensive nature and ability to capture the multifaceted implementation aspects in health care settings. By employing the CFIR, the analysis systematically examined various factors influencing the implementation of the PAEHR system, providing a holistic understanding of implementation. The information under the different domains was thereafter inductively analyzed using content analysis according to Graneheim and Lundman [41]. The information was coded, and the codes were grouped into categories under the theme of each CFIR domain. Feedback from participants was not collected. The analysis was conducted using NVivo 1.4 (Lumivero).

According to the CFIR, the following five domains are essential for the implementation of interventions in health care: (1) characteristics of the intervention (eg, adaptability and complexity), (2) inner setting (eg, structural characteristics and culture), (3) outer setting (eg, patient needs and resources; cosmopolitanism), (4) characteristics of the individuals involved (eg, knowledge and beliefs), and (5) implementation process (eg, engagement, evaluation, and reflection).

The CFIR combines different theories and provides a comprehensive framework for implementation research. The different domains are further described below in the results section.

Consolidated Criteria for Reporting Qualitative Research (COREQ) guided the reporting of this study [42].

### Ethical Considerations

The study received ethical approval from the Stockholm Ethical Review Board (2017/1028-31). All study participants provided written informed consent before participating in the study. In accordance with the requirements of the Swedish Ethical Review Authorities, participants were informed that their participation was voluntary and that they could choose to revoke their participation at any time. Transcribed interviews were deidentified to protect the participants’ privacy, and confidentiality was preserved when reporting the results. No compensation was given for study participation.

## Results

Fourteen interviews were conducted with a mix of HCPs, including a counsellor, dietitians, physicians, physiotherapists, medical secretaries, registered nurses, and an occupational therapist (Table 1). The ages of the participants ranged from 27 to 67 years, and their work experiences ranged from 5 to 36 years.

**Figure 1:**
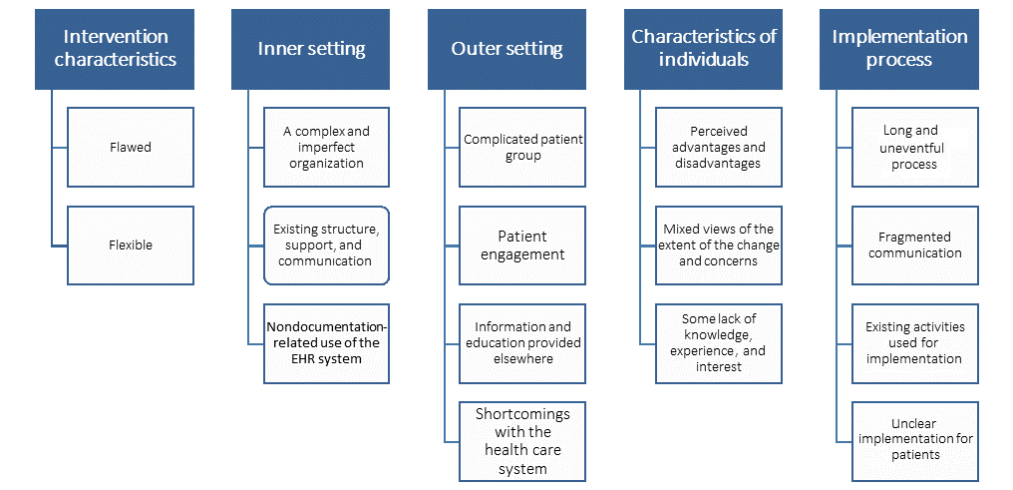
Themes that emerged under the Consolidated Framework for Implementation Research dimensions. EHR: electronic health record.

The analysis of the 14 interviews resulted in 15 categories under themes that were equivalent to the 5 CFIR domains (Figure 1). The categories have been further described below, with any subcategories italicized.

**Table 1:** Participant characteristics.

Characteristic		Value (N=14), n (%)
**Profession**		
	Counsellor	1 (7)
	Dietician	2 (14)
	Medical secretary	3 (21)
	Nurse	2 (14)
	Occupational therapist	1 (7)
	Physician	3 (21)
	Physiotherapist	2 (14)
**Age (years**)		
	<39	1 (7)
	40-49	5 (36)
	50-59	3 (21)
	≥60	5 (36)
**Experience (years)**		
	5-9	3 (21)
	10-19	6 (43)
	20-39	5 (36)

### Intervention Characteristics

In the CFIR, intervention characteristics refer to the attributes of the intervention itself, including intervention source, features, complexity, and adaptability. In our case, the intervention was the PAEHR system that made EHRs accessible to patients. Our study uncovered two categories: (1) flawed and (2) flexible. These are mainly related to the concepts of complexity and adaptability in the CFIR domain.

#### Flawed

The HCPs voiced concerns regarding various shortcomings within the system, pointing to complexities in usage, challenges with terminology, and limitations in safeguarding sensitive patient information. The EHR system, from which information is shown to the patient through the PAEHR system, was perceived as complicated. Templates used to structure the documentation were perceived as challenging; hence, *writing the records was difficult*. Moreover, patient ORA caused uncertainties regarding medical terminology use.

It would have been good for patients if the record’s templates looked the same, and for us too. I mean, the record’s templates are difficult to follow sometimes. It's a lot to fill in and a lot to klick to fill in and sometimes it is forgotten.

Dietician

Additionally, the HCPs expressed that the PAEHR system *lacked flexibility* and *safeguarding features*, particularly for mental health patients. Some HCPs were worried that patients could still see if information was hidden, and 1 HCP felt reluctant to document suspicions or early hypotheses as a consequence.

In contrast, some HCPs would have liked more information from the EHRs to be accessible to patients and to be able to monitor patient activities in the PAEHR system. 

#### Flexible

The HCPs highlighted features that would enable discreet communication between HCPs, options for some information blocking, and allowances for incomplete records not to be shown to patients for up to 14 days.

[…] we talked about the existence of the hidden part, where one could write where they [the patients] could not see.

Dietician

The HCPs also reported being aware of previous implementations of the PAEHR system, with favorable outcomes.

We were informed that Uppsala {another region} had also used it and that they were happy.

Physician

Moreover, the PAEHR system was perceived to be *more confidential* than paper copies. For example, for patients experiencing domestic violence, it reduced the risk of paper copies being accessible to others.

### Inner Setting

According to the CFIR, inner setting refers to the organization’s internal environment where implementation takes place. It encompasses the organization’s structural, cultural, and political aspects [23]. In our case, it refers to the PCC where the HCPs worked. Three categories emerged under this domain: (1) a complex and imperfect organization, (2) existing structure, support, and communication, and (3) nondocumentation-related use of the EHR system.

#### A Complex and Imperfect Organization

The HCPs described a *challenging work environment*, with varying tasks and a high workload, where they constantly discuss priorities. They mentioned that reflection, education, and documentation are not prioritized.

We must increase the number of visits, we can't go to education since it takes time. There is a desire to be better and produce more simultaneously. There is no common sense and no regard for the staff, it should just move faster and faster and people can't keep up.

Physician

According to the HCPs, they cannot *adequately prepare* for visits, which can upset some patients. They deal with *unpredictable visits*, *unclear/unsolvable conditions*, and *complicated treatments.* Assessments can be complex, affecting the clarity of notes. Moreover, the visits are short, and there is a lot of knowledge and information transfer, requiring confirmation of the understanding of patients. In cases related to children, they may be required to rely on information from parents. Misunderstandings can thus occur.

In addition, *communication with patients was restricted.* Certain information (eg, test results) was supposed to be communicated by the responsible physician, which hindered other HCPs (eg, medical secretaries and nurses) from answering patient questions. Moreover, requests to make changes to the record must pass through the manager.

Further, some HCPs had *dealt with threatening, aggressive, and rude patients* and were fearful. Moreover, the HCPs were repeatedly questioned about the records by their manager and patients. Patients could question slightly irregular but otherwise typical test results, and explanations could be time-consuming. Additionally, the HCPs avoided documenting suspicion and were uncertain of how safeguarding functions, such as hidden keywords and hiding unsigned records, work.

Sometimes you get aggressive patients that yell and act badly […] we are exposed to a lot. If for example there is only 30 slots today but there is a lot of people calling.

Registered nurse

Furthermore, the HCPs *had encountered several issues* when caring for patients, and these were related to the systems they use in general. Serious diagnoses had mistakenly been revealed to patients, referrals and reports had disappeared, and lab orders had remained active despite completion. Their IT systems were described as ineffective, difficult to use, and annoying.

The HCPs also reported a *lack of unification in documentation practices.* Physicians dictate and others transcribe the notes, while other HCPs write their notes themselves. Some respondents wrote on notepads first. The length of the notes varied, often with more junior HCPs writing more comprehensive notes.

The HCPs also described issues with the *record’s quality*. Errors and gaps could occur during transcription due to the transcriber’s lack of experience. Substandard language could also be used. Moreover, patients have reported errors in their records, and the HCPs were uncertain if the errors were corrected. According to 1 respondent, corrections are seldom made.

Further, *documenting and transcribing on time were challenging*. Some HCPs reported documenting after every patient or on the next day, and some dictated their notes with patients in the room. Additionally, *unsigned records were problematic* for the management. Transcribed notes are not always reviewed due to time constraints, and unsigned notes may contain blanks and errors. According to 1 HCP, some HCPs simply refuse to sign.

That's something that is constantly being pointed out, that we have to sign the records. [Counsellor] 

#### Existing Structure, Support, and Communication

The HCPs reported *some documentation structure.* They have always been conscious of their writing but might be more restrictive now. The transcribing medical secretaries have always strived to write comprehensively, and this is something they learned during their education.

Moreover, some HCPs have always *strived to document and sign records promptly*. Some HCPs had received directives to document quickly to ensure nothing lingers. Further, according to 1 HCP, HCPs have time allocated for administration, which should enable them to complete the tasks. Certain registrations were also required for financial reasons.

I usually sign them immediately because I know they will linger on a list which will be difficult to sign afterwards.

Registered nurse

Additional structures include functionality for tracking the people accessing the records. Sometimes, HCPs must have the patient’s consent to view the records. There are also routines for providing patients with paper copies and dealing with upset patients, as well as channels for communication between nurses and physicians related to patient requests. 

Furthermore, in relation to the implementation of new services, there is an organizational desire to change and improve, and the HCPs had previously participated in pilot projects.

Moreover, *support for documenting is provided* in different stages. HCPs might point out significant errors to each other. Transcribers, who are often medical secretaries, point out or alert physicians to errors or missing information in the records. At times, they may correct the errors and inform the physician. Moreover, medical secretaries assist physicians with insurance-related documentation. Furthermore, unsigned records are tracked by managers, who remind HCPs to sign.

I try to reconstruct what the physicians have dictated, I try to reconstruct it into a more understandable language for patients.

Medical secretary

Further, a *structure for communication with patients about their health care exists*, but it varies among HCPs***.*** Moreover, there is *established internal communication* among HCPs through regular discussions, seminars, and education about eHealth, which are led by HCPs at the PCC.

#### Nondocumentation-Related Use of the EHR System

Additionally, a few HCPs reported using the EHR system as an alternative communication channel among HCPs. HCPs could send internal letters to each other.

### Outer Setting

In the CFIR, outer setting refers to the external context surrounding the organization where the implementation occurs. It includes patient needs and resources, external policies and regulations, and the broader sociocultural and economic environment. We have therefore included categories related to patients’ needs and resources in this theme: (1) complicated patient group, (2) patient engagement, (3) information and education are provided elsewhere, and (4) shortcomings with the health care system.

#### Complicated Patient Group

According to the HCPs, their *patients are heterogeneous* with varying levels of knowledge and engagement in their care. While some patients are *good at scheduling appointments and understanding*, are *curious*, and *show interest* in their health records, HCPs also encounter patients who do not cooperate and who misinterpret, overanalyze, and get stuck. Parents who come in without their children and patients who are worried, confused, or forgetful (losing papers and copies) can be unreachable. The HCPs reported having a mix of new patients and patients they meet often and know well. Many of their patients are old, and some are immigrants with difficulties with the Swedish language. These groups were perceived by our respondents to be less likely to read their records online. Moreover, 1 HCP believed that only a few people read their PAEHRs, with readers being those engaged in their care. The PAEHR system was described as inaccessible for some and thus was described as excluding. Moreover, the HCPs thought that patients would only be interested in reviewing their records in special cases. In addition, while there were many requests for printed copies, few were collected.

Further, according to the HCPs, the PCC’s patient population has *substantial health care needs*, which the available care cannot meet. Patients were described as frustrated over the lack of accessibility. Some HCPs acknowledged the difficulty for patients, both in reaching the health care center and managing the information provided during the visit.

We have patients waiting for an appointment who haven’t gotten to meet a physician. ‘But you were here the day before yesterday…’ and they say ‘But now it is about this issue…

Registered nurse

Moreover, according to the HCPs, patients have *high expectations* and are often unsatisfied. The HCPs stated that there was an expectation to remember everything, have answers to all questions, and read the records before a meeting, or they may be perceived as unprofessional. Unpreparedness can upset patients. The HCPs also described unsatisfied patients who perceive HCPs as incompetent and may want to change or include notes in the record and request a second opinion, with some wanting to change health care centers. Some patients were described as being controlling.

Furthermore, according to the HCPs, some patients lack necessary technical equipment like a printer, a telephone, and personal electronic identification (eID), as well as knowledge about the use of the internet.

#### Patient Engagement

The HCPs reported working in a patient-centered way and *engaging patients in their care*. They aimed to engage patients in shared decision-making and encouraged patients to, for example, follow-up and prepare questions before their meetings. The HCPs also reported engaging patients during the meetings by showing them their records and encouraging them to use the PAEHR system when copies are requested. The HCPs also described how they engage patients in the documentation by confirming their notes with the patients and mentioned that they may read and explain part of the records to the patients. In addition, HCPs could meet with patients for a follow-up earlier if requested.

I have done it for many years, asked patients to write down things they are wondering about and they can also bring up things they have read.

Counsellor

#### Information and Education Provided Elsewhere

Some HCPs reported learning about documenting PAEHRs at their previous work or during their education. One HCP also recognized similarities to another implemented initiative called SVF (standardized care process), where HCPs must document findings quickly.

#### Shortcomings With the Health Care System

A few HCPs expressed *perceived shortcomings with the health care system*, including a lack of reimbursement, misprioritization, and unnecessary care processes.

### Characteristics of Individuals

In the context of the CFIR, characteristics of individuals refer to the personal attributes, attitudes, and behaviors of individuals directly involved in the implementation. This domain considers their knowledge, skills, beliefs, and motivations related to the implemented intervention. In our case, the individuals are the HCPs, and we have included their more general opinions on patient ORA and the use of PAEHRs in health care. Three categories emerged under this theme: (1) perceived advantages and disadvantages, (2) mixed views of the extent of the change and concerns, and (3) some lack of experiences, knowledge, and interest.

#### Perceived Advantages and Disadvantages

The HCPs stated both the advantages and disadvantages of the PAEHR system. While some HCPs thought it was essential, others were ambivalent. Some had become more positive over time, while some remained negative. Some HCPs explicitly expressed that they were against ORA, while others were described as angry by their colleagues.

The HCPs perceived that the implementation of the PAEHR system was *patient-driven* and that it was at the request of patients. There was also the perception that the records are the patient’s property and that the PAEHR system is the patient’s right and beyond the control of HCPs. The HCPs also thought that it was part of a general trend toward more transparency in health care. One HCP reflected that the lack of understanding of the need for patients to read their records may be due to their old-fashioned views. Moreover, the PAEHR system was believed to increase access to records and participation and to engage patients. Further, the HCPs thought that patients accessed the records for many reasons: they were interested or curious, they wanted to follow-up, they wanted to remind themselves about the findings, they wanted to improve their understanding, and they desired to participate in decision-making. Some HCPs thought that some patients read their records because they were worried or because they were suspicious and wanted to see what was not said at the visit.

I think it is good for openness that it isn't a closed document, that they should be able to read {the record} without asking for it.

Dietician

In health care in general, the HCPs thought that the PAEHR system *increased health care quality*. It made them more reflective, clearer, more precise, and more direct with their assessments. The PAEHR system was also described as a reminder to write the notes on time, and patient understanding was thought to be more critical than using medical terms. Moreover, the PAEHR system was considered environmentally sustainable since it reduces the number of paper copies and letters sent. The HCPs perceived the PAEHR system to be beneficial for transparency. According to some HCPs, reading the records is important since it concerns the individual’s health and is essential for patients to follow their care. They also perceived it as time-saving for patients and a way of making the information more accessible, especially for patients experiencing difficulties due to disability. Moreover, the PAEHR system was seen as a control function, where patients can see that the most important information is included and can make sure agreed-upon actions are being taken, such as sending referrals. The quality of documentation and the quality of care could potentially be improved if patients could point out errors. The HCPs also hypothesized that increased accessibility of the records would increase the likelihood of use among patients.

In terms of gains I don't know but the advantages in terms of me thinking a little extra of what I am doing in relation to patients. That I think about it so that it is clearer maybe in general.

Counsellor

For themselves, the HCPs saw a *potential to lighten the workload* of writing summaries and reduce calls from patients. The HCPs could refer to the PAEHR system if paper copies of the records were requested. Moreover, once patients read the reports online, they might not see the need to book a face-to-face visit, reducing unnecessary visits. They also saw the potential of using the PAEHR system as a treatment tool to clarify treatment plans and goals.

For patients, the HCPs saw the potential for the PAEHR system to *support participation and collaboration* through increased availability and accessibility. With the PAEHR system, patients could have information from a visit in writing, which could support understanding. This was especially important since the visits could be emotionally charged for some patients, making it hard to retain information and resulting in misunderstandings. According to 1 HCP, it is also a way for patients to see that something comes from the visit. The HCPs noted an opportunity for making treatment plans clearer for patients and potentially increasing compliance, and they wished that patients would read the reports in preparation for a visit. This could potentially reduce patients’ worry and increase their confidence in health care.

I think a positive effect can be that patients may get a little control over their record and can look. We write what we have done and they follow the test results.

Physician

Additionally, the HCPs saw potential for increasing patients’ involvement in their care by, for example, tracking medication changes and test results, and for facilitating patients’ understanding of how their behaviors may influence their health. Further, the PAEHR system was perceived to be beneficial for families of older patients and patients with different health care providers as it gathers all information in one place. In addition, ORA was perceived to be time-saving and cost-saving, improving patients’ response times when issues arise. According to some HCPs, the service was also easy to use. However, the PAEHR system was not purely positive according to some HCPs.

I think there are both pros and cons. If you are hypochondriac it can also be a risk. If you get more information than you need.

Medical secretary

According to the HCPs, *records have other sometimes contradictory purposes*. On the one hand, the HCPs perceived patients as the owners of their records, while on the other hand, the records were considered a professional tool and thus must be detailed. The records could include information that patients might not want on record and assessments that patients might disagree with.

Moreover, the HCPs argued that the records could contain confidential and sensitive information and that not all information is meant for the patient. Documentation is a way to reduce the cost of, for example, test duplication, and to be able to protect other patients from, for example, infections. Moreover, documentation is needed since patients may not be entirely honest about their previous health care contacts. 

Further, some HCPs saw *limited benefit* with the PAEHR system or even considered it an *unnecessary* service. A few HCPs were uncertain of or did not see the importance of patient ORA. They thought that better solutions are present to engage patients in their care and argued that they keep the documentation in the best interest of patients, making it unnecessary for patients to access it. They could not see the benefits of the PAEHR system, as they believed that notes from the PCC might be basic, stressing that patients could already request copies of their records on paper if needed. They believed that health outcomes and personal contact between the HCP and patient were more important and that too much focus was being placed on EHRs. Moreover, they believed that the PAEHR system was unnecessary as their previously experienced issues with unsent referrals, which PAEHRs might have resolved, had already been addressed.

I don't think benefit from reading what the physician has written or what that test results show. If they are very interested they can ask.

Physician

Moreover, according to the HCPs, the PAEHR system was *not optimal*. They believed that the records contained little information, which might not be visually optimal. The information provided might also not be essential or summarized for patients. In addition, documentation was not standardized. Further, the HCPs thought that the service should be restricted to signed records and access should be delayed. Moreover, they thought that the platform was difficult to navigate.

Additionally, the HCPs believed that the PAEHR system was *unavailable or inaccessible to some patients*. They believed that most users would be younger, while elderly people, people with dementia, and migrants with language difficulties would be excluded, creating a double system. They also believed that patients with low health or digital literacy would be excluded and that the use of the system would depend on the patient’s illness.

I think the day I myself get dementia and sitting there struggling with all the passwords, It would be horrible and you cannot keep that organized.

Physiotherapist

Further, some HCPs believed that the PAEHR system would be a *disservice to patients.* It would give a false sense of control, increase worry, and be misinterpreted, increasing HCP workload with additional calls and questions. One HCP speculated that patients might read their records to find things to get sick over. Additionally, it might be unpleasant for patients to read serious diagnoses by themselves. According to 1 HCP, it might also be *difficult for patients to understand* the content without a medical education, and additional visits would be needed regardless. According to these HCPs, an oral explanation would be ideal, addressing additional uncertainties during a visit. One HCP did not think patients should read their records at all and stated that patients with severe mental health issues might be negatively affected. According to 1 HCP, the risk depends on the person.

#### Mixed Views of the Extent of the Change and Concerns 

According to some HCPs, the implementation of the PAEHR system meant *little to no change*, potentially since the introduction was so recent that any impact was yet to be seen. Some HCPs were *unmoved* by the implementation. They did not believe the PAEHR system was meant to change care delivery or affect documentation due to, for example, fear of repercussions from patients. According to them, everyone knew it was coming long in advance and they were prepared.

Moreover, *few documentation requirements were new, and the HCPs therefore made little to no changes* to their practice. The HCPs reported that they have always been reflective when writing and tried to be careful and precise. They wrote in a way that the records would be readable for patients. Some HCPs reported that they always wrote and signed the records quickly. Moreover, the HCPs always discussed the notes with patients. The difference now was that the records were online.

What I have thought a little more about is writing the notes on time but that is something that should be done anyway so it is not really new. But it is a reminder to write more understandably which also is not something new, just a little extra.

Occupational therapist

In contrast, some HCPs thought that *the PAEHR system would require a change* for those HCPs who had not previously been educated about patient documentation. This could be especially true for more senior HCPs who often use medical terms and abbreviations. Some HCPs thought that it would take time to get accustomed to it and would be time-consuming since it was unfamiliar. One HCP reported that they now try to avoid Latin words, which requires effort.

#### Concerns

Many of the previously mentioned issues concerned HCPs, worrying them and making them skeptical. Some HCPs were skeptical as a patient as well. Although much had not turned out as they feared, there were still concerns that effects might come later. One HCP expressed that the novelty of the PAEHR system was scary.

The HCPs worried about *potential consequences*. They feared potential increases in criticism, questioning, and being reprimanded. They believed that their workload would increase, especially for physicians, due to patient calls and questions. Furthermore, they believed that the system could affect the health care center’s reimbursement. In addition, the HCPs had *many thoughts and questions about implementation* and the related practicalities. The increased demands for clarity and timeliness of documentation were causes for stress due to the existing time pressures in their work. Furthermore, the HCPs were concerned that the documentation changes would make the notes less understandable. One HCP felt unprepared.

It was more of how it going to happen. What would happen if patients read, are they going to scold us …will they get mad over the information they have gotten.

Registered nurse

Further, the HCPs *worried that patients might be negatively affected.* They were worried that the records would upset their patients and that the patients would not like or agree with the contents and would shop around for care. This was particularly concerning in regard to children (eg, social service reports could potentially influence children’s confidence in their providers). They were also worried that reading the records could affect patients’ confidence or trust in their providers or the treatment. Moreover, the HCPs were concerned about patients who were unable to ask questions or get support from a provider. They believed that the system could cause unnecessary worry. They thought that the records might be especially difficult to understand or interpret for patients not working in health care, which might lead patients to search for information on Google. One HCP suspected that some patients had concealed an appointment upon reading the records, which was concerning. Patients also felt anxious because they did not know what was being written about them.

Moreover, the HCPs were *concerned about the security* and integrity of patients, the preservation of doctor-patient confidentiality, and unauthorized access to records.

However, the HCPs also expressed that things *did not turn out as they had thought or feared.* Patients were not reading their records before being contacted, calls to the PCC did not increase, and they were not reprimanded by the management. They had not made any of the changes that they thought they would need to make. According to 1 HCP, everything had solved itself. 

#### Some Lack of Knowledge, Experience, and Interest

There was *little to no knowledge about the functions of the PAEHR system* among the HCPs. Some HCPs were unaware or unsure of what patients could view. One HCP wished that they had insights into implementation experiences from other regions.

I know they can see the notes, test results and referrals I think, but not for example biopsies and such, I don’t think they can see that, and not Xrays.

Physician

Furthermore, the HCPs had *little to no knowledge about the service’s benefits and effects on patients*. Patients’ experiences and the number of patients accessing their records were unknown. A few HCPs had second-hand information about patients’ experiences. According to some HCPs, patients appeared not to be reading their records and should be reading more. According to 1 HCP, few patients seemed to use online services in general, with 1 HCP speculating that patients in specialist care might be more interested.

Some HCPs did *have personal experiences* reading their own records or a family member’s records online or on paper. Those with some ORA experience had just tried it once, and some of their records were empty. A few of them had read their records at the doctor’s office out of interest, whereas some had requested paper copies of their hospital records. One HCP had experience with other services on the patient portal, and a few HCPs explicitly expressed disinterest in the service and in reading their records.

I have been in mine and apparently I have nothing there. But I have looked with my husband at his and then everything was actually correct.

Registered nurse

### Implementation Process

According to the CFIR, implementation process refers to the methods, strategies, and activities used to facilitate the adoption and integration of an intervention into a particular setting or organization. This domain examines how the intervention is introduced, implemented, monitored, and evaluated over time. Four categories emerged under this theme: (1) long and uneventful process, (2) fragmented communication, (3) use of existing activities for implementation, and (4) unclear implementation for patients.

#### Long and Uneventful Process

According to the HCPs, information was provided, but the time until the service was available appeared *outdrawn* for some. It had been ongoing for years, and some HCPs had to be reminded that it was ongoing. According to 1 HCP, many had grown tired of nothing happening. Making the services available was, however, quick and smooth, and suddenly, it was there overnight. The long implementation process reportedly led to some loss of motivation to act.

The negative is that it had been talked about for so long but it never got started.

Physiotherapist

#### Fragmented Communication

HCPs were *partially informed*, discussions occurred, and some participated in educational activities. According to 1 HCP, they had a seminar about 1 year before the implementation of the PAEHR system. Some HCPs recollected an education day for some professions, where they learned about and looked at the service. They, however, received no information about when and how the PAEHR system would be launched. According to the HCPs, they did not discuss the technical aspects of the service. One HCP reflected that maybe they did not need more information, while another thought that a user video would have been beneficial.

I have no idea how it was done […] we just got information that it going to be online. Nothing more.

Physician

Additionally, the scope of information about different aspects of the implementation varied. According to the HCPs, there were discussions about work processes, safeguarding functionalities, how to write, and flexibility aspects, and the importance of completing the records on time was also emphasized. According to 1 HCP, they were told to “do as they do.” Furthermore, the HCPs reported receiving information about experiences from other implementations and having some follow-ups at their usual meetings, according to individual accounts.

#### Existing Activities Used for Implementation

According to the HCPs, information related to the implementation of ORA was provided during their existing meetings related to eHealth. Information was also provided by the usual leader of these meetings, who was one of the HCPs at the center.

#### Unclear Implementation for Patients

The HCPs were uncertain about how patients were informed, and according to some HCPs, *patients were uninformed*. The HCPs had not noticed any major information campaign targeting patients. Some thought that HCPs were supposed to inform patients, and some occasionally did so. Those informing patients mainly did so with patients who showed an interest. One HCP reported now informing patients more frequently (6 months after implementation). Moreover, there were reports of some brochures in the waiting rooms. There were attempts to encourage physicians to inform patients.

[…] if they got something in their mailbox, I do not know but I don't think so.

Medical secretary

## Discussion

### Principal Findings

Although patients in Sweden have had ORA since 2018 (the first implementation began as early as 2012), few studies have focused specifically on implementation in primary care from the perspective of HCPs. To our knowledge, this is the first comprehensive study of the implementation of PAEHRs in Swedish primary care from the perspective of a diverse group of HCPs. Our study identified several potential barriers and facilitators related to PAEHRs, the primary care setting, and patients’ and HCPs’ characteristics, as well as barriers and facilitators related to the implementation process (Table 2). We will discuss the identified barriers and facilitators in relation to previous research.

**Table 2:** Potential barriers and facilitators related to patient-accessible electronic health record implementation.

Domain	Barriers	Facilitators
Intervention characteristics	Shortcomings of PAEHRs^a^	Flexibility of PAEHRs
Inner setting	A complex and imperfect organization Nondocumentation-related use of the EHR^b^ system	Existing structure, support, and communication
Outer setting	Complicated patient group Shortcomings with the health care system	Patient engagement Information and education provided elsewhere
Characteristics of individuals	Perceived disadvantages of PAEHRs Mixed views of the extent of the change and concerns Some lack of experience, knowledge, and interest	Perceived advantages of PAEHRs Mixed views of the extent of the change
Implementation process	Long and uneventful process Fragmented communication Unclear implementation for patients	Use of existing activities for implementation

^a^PAEHRs: patient-accessible electronic health records.

^b^EHR: electronic health record.

### Barriers for the Implementation of PAEHRs

The HCPs reported several shortcomings with the PAEHR system. Complexities in usage, challenges with terminology, and a lack of safeguarding features could be potential implementation barriers. Complexities in EHR system usage might explain the reported lack of unification in documentation practices, the poor quality, and the difficulties with accomplishing and signing the records, further explaining the related concerns. Challenges using EHR systems have previously been described [43,44], and documentation for patients is an additional challenge where support is needed. This reflects complexity, which is one of the intervention characteristics that influence implementation according to the CFIR [23]. Additionally, adaptability influences implementation according to the CFIR, and the PAEHR 1177 journal lacked flexibility regarding sensitive information according to the HCPs. However, the definition of sensitive information by the HCPs might need to be considered together with that by patients, as the limitation of information in the PAEHR system has previously been considered problematic by patients [45]. The improvement suggestions by HCPs have been related to these described issues [39]. Many related improvement suggestions have aligned with patients’ experiences, but suggestions related to the restriction of information have not aligned, and patients want access to more information [46]. Enhancing the usability of the PAEHR system and providing support and education are potential solutions to these barriers. 

The complexity of the health care organization and its imperfections are other potential barriers. The strained working environment has been raised as a cause for concern by HCPs in both specialized and primary care [14,47,48]. Further, the HCPs described several potential barriers related to documentation practices, that is, a lack of unification in documentation practices, poor quality, and difficulties documenting on time and signing the records. In a recent survey of GPs in the United Kingdom, the majority believed patients would find errors in their GP records [8]. Swedish patients have also reported finding errors in their records [49]. Addressing system-level issues within the primary care system by, for example, increasing resources and training HCPs might reduce these barriers.

HCPs’ descriptions of their patient groups as challenging might explain their concerns about the negative impacts on patients. It has previously been reported that patients potentially do not understand their records [12-14], and patients have had similar concerns [50] and have reported difficulties understanding their records [51]. However, some research suggests that most patients understand most of the information available in their PAEHRs [20,52].

The potential exclusion of some patient groups has also previously been described [19,20] and needs to be addressed. Experts have recommended education or support for patients in using PAEHRs as a core part of the implementation of the PAEHR system [32].

According to the CFIR, beliefs and attitudes toward the intervention impact implementation [23]. Resistance among HCPs has been previously highlighted as the main barrier to PAEHR implementation [19]. According to the HCPs in this study, one of the perceived disadvantages was that the purpose of an EHR might not align with the purpose of a PAEHR, which has previously been highlighted [13,14]. As previously mentioned, the HCPs were unsure what needed to be changed. When ORA was first introduced in Sweden, the assumption was that this would not require substantial changes to HCPs’ work processes [31], yet this is not the experience of all HCPs. Addressing this ambivalence with guidelines on how ORA will impact HCPs’ documentation practices could overcome some of the experienced resistance.

Another disadvantage was that the HCPs perceived that there was little to no benefit of the PAEHR system. This could reflect a lack of existing research at the time of interviews or simply a lack of communication of existing evidence to HCPs. Current research shows several benefits for patients [6], with minorities and elderly people reporting more significant benefits from ORA [20]. Whether the benefits are sufficient to motivate the introduction of ORA also seems to be questioned by HCPs.

The mixed views on the extent of the changes ORA entails highlight the previously mentioned ambiguity regarding the service. These mixed views might explain the perceived disadvantages expressed by some respondents, and as previously mentioned, the purpose of PAEHRs needs to be clarified. Regarding documentation, there have been several reports of it changing due to the implementation of ORA [12,16,17,53-55]. However, HCPs have also previously reported not experiencing all they feared [13,16,55].

Furthermore, HCPs and other stakeholders have previously expressed several of the concerns raised [13,31]. Studies have shown some negative impacts on HCPs’ work environments in terms of increased demand and loss of control, as well as some positive impacts in terms of increased support [12,16]. Moreover, patients have expressed a fear of being worried [50]. According to a Swedish national patient survey, 26% of patients had been worried by information on their record, and they commonly called or raised their concerns at subsequent visits with their providers [18]. Furthermore, concerns regarding privacy and security have previously been raised [13]. Patients also have these security and privacy concerns, but to a smaller extent, and have not experienced many breaches [20,21,49]. Additionally, patients have mainly reported improvements in patient-provider relations due to ORA and have not reported worsening of the relations [21].

A lack of experience with the service among some HCPs could be another potential barrier, as previous research has found that HCPs with experience in using PAEHRs are more positive toward the service [56]. Exposing HCPs to the PAEHR system could thus potentially have a positive impact on its implementation.

The long and uneventful implementation process and the fragmented communication are potential barriers and might explain the lack of knowledge, experience, and interest among our respondents. In a study about PAEHR communication reach, HCPs (eg, nurses and physicians) received information from different sources, and despite several efforts, 7% of the HCPs had not received information at all [57,58]. A method for evaluating information reach might be a potential solution to this issue.

A more effective process for communication about delays could also have been beneficial. HCPs in other settings have requested more detailed information and more involvement in implementation [57]. More and timelier information about the service is thus a potential solution that could have mitigated some of the concerns.

Follow-up also seems to have been limited, which could have contributed to the concerns. According to the CFIR, reflection and evaluation are essential implementation activities [23]. More continuous discussions about the implementation of PAEHRs could have supported the implementation.

How the PAEHR system was introduced to patients was also unclear and could be related to the fragmented communication. There are reports of patient involvement in the development of the PAEHR system, but these efforts were minimal during regional implementation [7]. Better implementation activities for patients in terms of support and education in a more structured manner might mitigate HCPs’ concerns about the potential negative effects on patients. There is also an indication that education might support patient use [59], and it has been recommended by experts [32]. The involvement of both HCPs and patients has been identified as an essential factor for successfully implementing PAEHRs [7]. A clearer and comprehensive implementation process for both HCPs and patients is a potential solution to these barriers.

### Facilitators for the Implementation of PAEHRs

The HCPs acknowledged some system flexibility related to communication between HCPs, information blocking, and access to incomplete records, which could be potential facilitators. Flexibility is an influential intervention characteristic according to the CFIR, and further development of flexible software solutions enabling HCPs to closely monitor and control ORA could further facilitate acceptance and implementation.

The HCPs did report some existing structures and support for documentation, potentially facilitating implementation. The fact that there was some structure for communication with patients about their health care and some structure for documentation could have facilitated the implementation of ORA. By further strengthening these existing structures, perceived issues could potentially be mitigated and implementation could be facilitated. Outlining them could also be part of a readiness assessment of the inner setting.

Prior patient engagement could have fostered the perceived advantage of PAEHRs in supporting collaboration and patient engagement. The additional information channel provided by the PAEHR system might have been a facilitator since conventional channels have been proven inadequate [57].

The PAEHR system might also be a potential solution to some of the issues HCPs experience with patients. As highlighted by some HCPs, ORA can increase understanding and trust, and reduce workload. Moreover, HCPs have reported increased support in their working environment in patient interactions due to PAEHRs [16]. The HCPs did describe the PAEHR system as helpful for patients, and they mentioned that it could possibly improve health care quality, which is a potential facilitator. These views are supported by patient-reported benefits of PAEHRs, such as improved patient-doctor relationships, medication adherence, and reduced anxiety [6,12]. The reasons for reading, according to HCPs, also align with patient-reported reasons for reading [18]. These perceived advantages could be used to inform HCPs and change attitudes toward the service.

The use of existing activities might have been a potential facilitator. However, complementary activities are warranted as research demonstrates the lack of reach of existing communication channels [57,58].

We found substantial ambiguity and high variation in the opinions and experiences of HCPs regarding the implementation of ORA. HCPs’ views on the ability and readiness of patients to engage with ORA and their views on the purpose and usefulness of EHRs appear to affect their perceptions of the impact (positive or negative) ORA will have on their work. This can become a barrier or facilitator to implementation, depending on what narrative dominates a specific context. Understanding this relationship is essential to adapt implementation activities and communication strategies to HCPs’ experiences.

### Strengths and Limitations

The recruitment of HCPs from a single PCC, which can cause center-related bias, was a limitation. The inclusion of HCPs with different professions, which can provide multiple perspectives, was a strength that reduced the risk of profession-related bias. Medical secretaries might not have the same accountability as other HCPs regarding the records, which might have influenced their views. However, since they play an essential role in the documentation process, their inclusion was a strength. While data saturation was not fully achieved, the information provided by the HCPs was rich, providing many valuable insights.

Conducting the interviews at the center might have influenced the findings from the interviews. Yet, this influence appears to be minimal, as the interviews generated a diverse set of views and opinions. Based on the qualitative nature of the study, it was not possible to determine the prevalence of the identified barriers and facilitators for ORA implementation. Further research with a quantitative study design is therefore needed.

On one hand, the use of an interviewer without an HCP background can be considered a strength since it mitigates bias toward the views of HCPs. On the other hand, there might be a loss of information and understanding due to a lack of experience in the health care setting.

Since the analysis was conducted several years after the interviews, new experiences and information might have influenced the analysis and interpretation. The analyzing author attempted to keep the analysis close to the interviews and was mindful of alternative interpretations, all while discussing uncertainties and emerging themes with the other authors, which can be considered a strength. Additionally, using the CFIR in the analysis might have mitigated some risk of bias but might have influenced the generated themes. 

### Implications

Health care systems can better support HCPs adopting PAEHRs, particularly in settings with limited resources and high patient loads, by addressing the barriers highlighted in this study. They could enhance EHR systems, address the challenges with the primary care setting, support patients, address HCPs’ concerns and knowledge gaps, and adopt clearer and comprehensive implementation strategies. Integrating patient experiences is also essential for improving PAEHR usability and its impact on care. Future research should examine systematic approaches, such as surveys, focus groups, and co-design methods, to ensure patient voices inform ongoing development and optimization. Exploring how PAEHRs can be integrated with emerging technologies to enhance coordination, personalization, and decision support presents another important avenue for future research and policy development.

While long-term engagement strategies were not the focus of our study, our findings suggest that ongoing support is crucial. Participants emphasized the need for open dialogue, shared experiences, and organizational backing to navigate the challenges of PAEHRs. These insights point to the potential value of sustained training, peer-support mechanisms, and clear communication from leadership as part of long-term implementation efforts.

While this study focused on Sweden, its findings may be relevant to other countries implementing PAEHRs. Many of the identified challenges, such as HCP engagement, documentation practices, and patient comprehension, are common across health care systems, regardless of national policies. However, variations in regulatory frameworks, EHR infrastructure, and patient access policies may influence how these findings translate to other settings. Future research could explore how similar challenges manifest in different health care models to further understand the global applicability of PAEHR implementation strategies.

Our study also revealed that some HCPs may alter or omit certain details in their documentation due to concerns about how patients might react, potentially affecting completeness. For example, the participants mentioned avoiding sensitive terms or adjusting wording to prevent misunderstandings. While we did not specifically evaluate the impact on clinical decision-making, these changes may have implications for the accuracy and utility of records. These issues have been explored in a recent scoping review [60], which also identified this as an area that warrants further investigation to understand the potential effects on care quality.

### Conclusions

This study underscores the importance of having a usable EHR system to support developments such as the implementation of ORA. It also emphasizes the importance of addressing organizational issues, such as the work environment in primary care, for optimal implementation of eHealth services such as the PAEHR system. This study also highlights the importance of HCPs’ views and experiences with their patients and their perceptions and attitudes toward the intervention in the implementation of services such as patient ORA. Additionally, this study stresses the importance of effective implementation processes and communication strategies for both HCPs and patients.

In summary, issues with the EHR system and organizational shortcomings need to be addressed for optimal implementation of ORA, where both patients and HCPs benefit and risks are mitigated. Furthermore, improved information and education for patients and HCPs are potential solutions to address many of the concerns and perceived disadvantages of implementing PAEHRs.

## Appendix

Multimedia Appendix 1 Interview guide for health care professionals.

## Abbreviations

CFIR: Consolidated Framework for Implementation Research
EHR: electronic health record
GP: general practitioner
HCP: health care professional
ORA: online record access
PAEHR: patient-accessible electronic health record
PCC: primary care center

## Data Availability

The datasets generated and analyzed during this study are not publicly available due to the qualitative nature of the materials and the terms of the ethical approval, but are available from the corresponding author upon reasonable request.
