# Supplementary material for: Health Care Professionals’ Perspectives on Implementing Patient-Accessible Electronic Health Records in Primary Care: Qualitative Study
Source: JMIR Med Inform. 2025 Jun 6;13:e64982. doi: 10.2196/64982 (PMC12181750; doi:10.2196/64982)
Supplement: Multimedia Appendix 1 [file medinform_v13i1e64982_app1.docx]

Multimedia Appendix 1. Interview guide for health care professionals.

Introduction

1. Gender
2. Age
3. Profession
4. Years in the profession
5. How long have you been working at this primary care center?
6. Do you know when the "Journal", i.e. patients' access to their medical records online, was introduced or began to apply to this primary care center? Did you work here when it was introduced?
   1. How did you experience the introduction of patients' access to their medical records online?
   2. What has been positive? What has been negative? Example...

*(implementation process, communication from management, training, technical support, etc.).*

1. Have you experienced any difficulties/challenges as patients have been given online record access? Give concrete examples!
2. Have you experienced any benefit/benefits from patients having online record access? Give concrete examples!
3. How well do you know the content and functions of the e-service "Journalen" that patients use to read their medical records online?
4. Have you ever read your own medical records?
   1. [yes] How did you read it? On paper? Through “Journalen” online? In the electronic health record you use at your workplace?
5. Have you used “Journalen” as a patient or relative?
   1. Please tell us about your own experiences with “Journalen”.

Questions about your work and medical records

1. When you meet a patient you have not met before, do you usually ask if he or she reads his or her medical records online/uses "Journalen"?
   1. [if Yes] Do many of your patients or their relatives read?
   2. [if No] Do you assume that the patient does NOT access their medical records?
   3. How often do you see the same patient several times?
      *[P] Is it possible to establish a relationship with patients/relatives?*
2. Do you recommend your patients to read their medical records online?
   1. Why/why not?
3. Do you remember what you thought about patients having online record access *before the introduction*?
   1. What do you think about patients being able to read their medical records online today?
4. Have you personally experienced that the e-service "Journalen" has affected your communication with patients?

*[P] Has it affected how well you understand each other?
[P] Do you get other types of questions? Has the language you use changed? For example, more or less medical terms...*

*[P] Changes in number of visits/phone calls/contacts via 1177*

1. Do you use your electronic health record in your communication with your patients during the patient visit? In what way.
   1. Why/why not?

**About specific visits/patient encounters**

1. Do your patients specifically refer to having read "Journalen" during visits/patient meetings?
   1. If yes –
      1. In which cases does this usually happen?
      2. Have they shown you their view of "Journalen" during visits/patient meetings?
      3. Do you have to explain words written in the record entries?
      4. Do they ask questions based on what they have read in "Journalen"?
   2. If no –
      1. Can you still tell if they have read "Journalen"? *For example, by using phrases from the record and bringing up information from the record without specifically mentioning it.*
2. Have you personally experienced that your patients prepare for visits by using "Journalen"?
3. If yes –
4. How do you know/notice it?
5. What do you think about that?
6. Does this affect communication during a visit/patient meeting?

**About patients who read their own medical records**

1. According to your own experience; What is the main reason for patients to read their medical records online?
2. Do you think it is important that patients can read their medical records online?
3. Do you know if your patients are accessing results/information that has not been signed?
4. If yes – what are your experiences with it?
5. Have you met patients who have received their first information about serious health problems by reading their records?
6. If yes –
7. How often does this happen to your patients?
8. How have patients been affected by this? Give concrete examples.
9. Are you familiar with the "early hypothesis" option, which does not appear when the patient reads medical records online?
10. Have you used it yourself?
11. Have you met patients who have misunderstood results or information they have read in “Journalen”?
12. If yes –
13. How often does this happen to your patients?
14. In what ways have patients misinterpreted information?
15. Have your patients pointed out errors in the medical records?
16. If yes –
17. Do you know how your patients found out about the errors?
18. Did these patients ask you to correct the errors or change something in the medical record?
19. How did you handle this?

**About more general consequences of the patient reading their own medical records**

1. Have you noticed any impact on the quality of care?
2. If yes – give a concrete example!
3. According to your own experience; Has the decision-making process [in terms of treatment, for example] been affected by the fact that patients can read their medical records online?
4. If yes – do you have a concrete example?
5. According to your own experience; Has the patients' online record access affected their adherence to prescribed treatment?

**About the impact on the work environment and tasks**

1. According to your own experience; Has the introduction of patients’ online record access affected your work?
2. If yes –
3. In what way?
4. Has your workload been affected? How?
5. Has the work process been affected? How?
6. Was your interaction with other healthcare workers affected?
7. Has the way you document information in the medical record been affected by the fact that the patient can access the notes online?
8. If yes – in what way?
9. In your opinion, what is the main purpose of your record keeping?
10. What is the main purpose of giving patients access to their medical records online?

**Specific about the log list**

1. Are you aware that patients in Uppsala can see a log list that shows who has logged in and read their medical records?
2. What would you think if the corresponding feature was introduced here?
3. What do you think about the fact that the healthcare staff's name appears in the log list?
4. Would it be beneficial for you to be able to see if a patient or family member (of the patient) has logged in to “Journalen”?

**Final Questions**

1. Do you have any suggestions on how patients' online record access can be improved to be more useful for you and your patients?
2. Is there anything we have missed to ask you about? Anything that you would like us to know about patients' online record access?
